# Immunotherapy in Acral and Mucosal Melanoma: Current Status and Future Directions

**DOI:** 10.3389/fimmu.2021.680407

**Published:** 2021-06-04

**Authors:** Lili Mao, Zhonghui Qi, Li Zhang, Jun Guo, Lu Si

**Affiliations:** ^1^ Department of Melanoma, Peking University Cancer Hospital and Institute, Beijing, China; ^2^ Global Medical Affairs, MSD China, Shanghai, China

**Keywords:** acral melanoma, mucosal melanoma, immunotherapy, immune checkpoint inhibitors, combination therapy

## Abstract

Acral and mucosal melanomas are extremely rare in Caucasians; however, they are the predominant melanoma subtypes in Asians and other non-Caucasian populations. Acral and mucosal melanomas share many clinicopathological features, including aggressive phenotypes, similar genetic landscapes, and grim prognoses. In spite of advances in melanoma management, patients with acral and mucosal melanomas show limited benefit from current therapies. The rarity of these subtypes of melanoma is a significant factor contributing to the poor understanding of these pathological subtypes and the lack of effective interventions. Furthermore, the mechanisms contributing to disparities between different types of melanoma remain largely unclear. Herein, we comprehensively review current knowledge on the clinicopathological characteristics and mutational landscapes of acral and mucosal melanomas, as well as providing an overview of current therapies for patients with these aggressive melanoma subtypes, focusing on available immunotherapeutic interventions. We also discuss pathological differences between different melanoma subtypes and summarize current knowledge on melanoma disparities between Asians and Caucasians. Finally, we discuss emerging immunotherapeutic strategies for the treatment of acral and mucosal melanomas, focusing on combination therapies with immune checkpoint inhibitors. Unraveling the unique features of acral and mucosal melanomas is key for their early diagnosis and for the development of effective therapies.

## Introduction

Melanoma is a type of skin cancer arising from melanocytes, the pigment-producing cells found in the epidermis, hair follicles, and iris, among other tissues. Although melanomas most frequently develop in sun-exposed areas of the skin (e.g., the chest, neck, back, and legs), they may also develop in the eyes and in parts of the body that are not exposed to the sun. Comprising only around 1% of skin cancer cases, melanoma is far less common than other types of skin cancer, such as basal cell carcinoma and squamous cell carcinoma (SCC) (1). In most countries, the incidence of melanoma has been increasing over the past decades (2). Factors behind this rise in incidence rates include increased implementation of skin cancer screening programs, increased UV exposure, the rising popularity of indoor tanning, an increased number of skin biopsies being conducted, improved public awareness regarding suspicious pigmented lesions, and an increase in the number of specialist clinics for melanoma (3–7).

According to the American Cancer Society’s estimates, approximately 106,110 new melanomas (31.91 per 100,000 person-years) will be diagnosed in the US in 2021 (8). In China, although the incidence of melanoma is lower than Western countries, it is growing at an annual rate of 3%–5% (9). The 2017 global burden of disease study revealed that the age-standardized rate of melanoma in China in 2017 was 0.9 per 100,000 person-years (10). Ancestral differences in the incidence rate of melanoma are particularly evident in the US, where populations are composed of individuals of diverse ancestral backgrounds. Calculations based on historical data from the US SEER database reported age-adjusted incidence rates of *in situ* melanoma per 100,000 person-years of 9.19 for non-Hispanic Whites, 1.26 for Hispanic Whites, 0.16 for African Americans, and 0.34 for Asians and Pacific Islanders (11). Furthermore, despite its rarity, melanoma is the leading cause of skin cancer-related death (8). The poor prognosis of melanoma is primarily due to the high metastatic ability of melanoma cells (12).

There are different melanoma subtypes that originate from various parts of the body and exhibit differential clinicopathological and histological characteristics. On the basis of the anatomical location and the degree of sun-induced damage, melanomas can be classified into four primary subtypes: skin melanomas without chronic sun-induced damage, skin melanomas with chronic sun-induced damage, mucosal melanomas, and acral melanomas (13, 14). Skin melanomas are collectively referred to as cutaneous melanomas (15). Acral melanomas account for around 1.0%-1.5% of melanoma cases in the overall US population, in whom the vast majority (over 90%) of melanomas are cutaneous (16, 17). In addition, mucosal melanoma is a rare melanoma subtype among Caucasians, accounting for only ~1%–2% of all melanoma cases (16, 18, 19). In sharp contrast, acral (42%–65%) and mucosal (20%–30%) melanomas are the predominant melanoma subtypes in China and in other Asian countries (11, 20–26).

Accumulating evidence suggests that epidemiological, anatomical, and clinical differences may exist among melanoma patients with different pathological subtypes (27, 28); disparities in the prevalence of pathological subtypes and risk factors among melanoma patients have also been reported (2, 29). In addition, treatment response and survival outcomes differ considerably among patients with different melanoma subtypes (30, 31). While the mechanisms underlying these disparities remain to be elucidated, genetic and environmental contributors are believed to exist (27, 32, 33).

Though causing significant mortality and exhibiting unique biological and clinical features, due to their relative rarity (compared with cutaneous melanomas) in Europe and North America, acral and mucosal melanoma are often overlooked and studies are scarce. Hence, our understanding of these melanoma subtypes remains limited, hindering the establishment of consensus on their optimal management. In this article, we review the current knowledge on acral and mucosal melanomas and discuss disparities in their clinicopathological features, mutational landscapes, tumor immune microenvironment, and treatment outcomes. We also summarize emerging treatment strategies, especially combination therapies with immune checkpoint inhibitors, for these rare melanoma subtypes. This comprehensive overview of acral and mucosal melanomas aims to facilitate the establishment of consensus guidelines on the optimal management of these rare melanoma subtypes, improve early diagnosis, and guide the development of more effective therapies based on their unique characteristics. All these are key to improving the survival of patients with acral or mucosal melanoma.

## Acral Melanoma

### Clinicopathological Characteristics

Acral melanomas, also known as acral lentiginous melanomas, develop on non-hair-bearing skin, including the palms of the hands, the soles of the feet, or under the nails (34). The clinicopathological characteristics of acral melanomas differ significantly from those of cutaneous melanomas, with acral melanomas being more aggressive than cutaneous melanomas, regardless of the ancestry of patients (35). Importantly, patients with acral melanoma tend to be older and have fewer atypical nevi and a lower incidence of sunburn than patients with cutaneous melanoma (26). Furthermore, ulcerations and thick lesions are particularly common among Asian patients with acral melanoma (11, 36, 37). Importantly, melanoma thickness (Breslow depth) and ulceration are among the most significant factors associated with poor survival outcomes (37, 38). However, the higher tumor thickness and ulceration rates in Asian patients with melanoma compared with their Caucasian counterparts may reflect differences in the clinicopathological characteristics of acral and cutaneous melanomas, such as the higher aggressiveness of acral melanomas (35). In a recent study of 1157 Chinese patients with acral melanoma, Wei et al. (39) showed that patient prognosis varied depending on the anatomical location of the primary tumor. The study also showed that initial tumor stage and ulceration were significant predictors of melanoma-specific survival. Intriguingly, patients with primary melanomas on the soles of their feet had a worse prognosis than those with primary lesions on the palms and under the nails (39).

Additionally, ancestry-related disparities in clinicopathological characteristics have been reported among patients with acral melanoma. The 5-year disease-specific survival (DSS) of 53.5% in Chinese patients with acral melanoma reported by Lv et al. (25) was considerably lower than the 5-year DSS of 70% in Caucasians reported by Bello et al. (40) and of 67% in a predominantly white US cohort reported by Behbahani et al. (41). Similarly, Bradford et al. (30) reported that, among different ethnic groups with acral melanoma in the US, the 5-year and 10-year DSS rates were low in Hispanic Whites (72.8% and 57.3%) and Asian/Pacific Islanders (70.2% and 54.1%), intermediate in Blacks (77.2% and 71.5%), and high in non-Hispanic Whites (82.6% and 69.4%) (30). However, these differences in 5- and 10-year DSS rates among the different ancestral groups were not statistically significant after adjusting for tumor thickness or stage (30). Wang et al. (11) reported that among Asians/Pacific Islanders, Black, non-Hispanic White, and Hispanic White patients with acral melanoma, Asians and Pacific Islanders presented the thickest tumors. Moreover, Bradford et al. (30) noted that acral melanoma diagnosis at stage III was more common in Asians and Pacific Islanders than in non-Hispanic Whites, Hispanic Whites, and Blacks. Collectively, the findings of these studies suggest that factors including advanced disease stage, ulceration, and high lesion thickness at diagnosis may contribute to the poor survival outcomes observed for Asian patients with acral melanoma.

### Mutational Landscape

As acral melanomas develop in sun-shielded areas of the body, they are not believed to be caused by exposure to ultraviolet (UV) radiation. Hence, oncogenic pathway activation in acral melanoma is UV-independent, in contrast to the induction of oncogenic circuits in cutaneous melanomas (42, 43). Whole-exome and whole-genome sequencing studies revealed that, compared with cutaneous melanomas, acral melanomas have fewer single nucleotide polymorphisms (SNPs) and indel mutations and a higher number of structural rearrangements and focal chromosomal copy number aberrations (44). Genes frequently mutated in cutaneous melanoma include *BRAF* (45%–50% of tumors), *CDKN2A* (13%–40%), *NRAS* (~30%), and *TP53* (15%–18%), while *BRAF* (10%–35%), *NRAS* (8%–22%), and *NF1* (11%–23%) are often mutated in acral melanomas (42, 45). However, the frequency of *BRAF* and *NRAS* mutations in acral melanoma is considerably lower than that in cutaneous melanoma, leaving most acral melanoma patients ineligible for treatment with BRAF inhibitors and the combination of BRAF/MEK inhibitors (13, 46).

A study of 514 acral melanomas revealed that alterations in the CDK4 pathway were frequent and that these alterations promoted G1 to S cell cycle transition and tumor progression. Notably, 82.7% of all samples harbored mutations in at least one of either *CDK4, CCND1*, or *P16* ^INK4a^ (47). mTOR mutations are also common in melanoma. Kong et al. analyzed 412 melanoma samples and found that 10.4% (n = 43/412) of the samples had nonsynonymous mutations in mTOR. Notably, mTOR mutations were more frequent in acral melanomas (11.0%) than in chronically sun-damaged melanomas (6.7%). They also found that mTOR nonsynonymous mutations were significantly associated with poor survival in patients with melanoma, suggesting that PI3K-AKT-mTOR pathway inhibitors may represent a promising treatment strategy for patients with mTOR-mutant melanoma (48).

Gene mutations affecting the MAPK pathway, including those in *C-KIT* and platelet-derived growth factor receptor alpha (*PDGFRA*), are more common in acral melanoma than in cutaneous melanoma (49). Interestingly, Dai et al. (50) found that the SNP rs2228230:T in *PDGFRA* resulted in lower PDGFRA expression levels and downstream signaling activity and was associated with favorable survival in patients with acral melanoma (50).

Additionally, compared with cutaneous melanomas [the predominant melanoma subtype in Caucasians] acral melanomas possess a higher number of chromosomal structural aberrations and copy number variations (CNV) and exhibit a low tumor mutational burden (TMB) (42, 45). Although ancestry-related disparities in the TMB have been reported for different tumor types, including lung cancer (51–53), it remains unclear whether Asian patients with acral melanoma exhibit lower TMB than their Caucasian counterparts.

In a multi-fluorescence *in situ* hybridization study, the frequency of *CCND1* amplification in 44 Chinese patients with acral melanoma was 45.4% (54). Mutations in telomerase reverse transcriptase (*TERT*), the gene encoding an enzyme crucial for telomere maintenance, also play a crucial role in melanoma development, with up to 50% of cutaneous melanomas harboring *TERT* promoter mutations (55, 56). However, a retrospective study of Chinese patients with acral melanoma showed that *TERT* promoter mutations were present in only ~5% of cases (57). In Caucasians, acral melanomas have been found to have a higher number of CNVs than cutaneous melanomas (42, 45). In contrast, in a Chinese cohort, a higher number of CNV amplifications were detected in non-CSD cutaneous melanomas than in acral melanomas (58). However, future molecular studies in patients with different ancestries are required to elucidate the relationship between ancestry and the mutational landscape of acral melanoma.

Data on the association between mutational profiles and response to treatment in patients with acral melanoma are relatively scarce. However, one recent study of 178 Asian patients with advanced melanoma (40% with acral melanoma) who received treatment with immune checkpoint inhibitors suggested that *NRAS* mutations, *TP53* mutations and *NF2* deletions are associated with resistance to checkpoint inhibitors, whereas *MYC* and *RPS6KB1* amplifications were observed more frequently in patients who responded to these treatments (59).

### Tumor Immune Microenvironment

The PD-1/PD-L1 axis is one of the most well-studied mechanisms contributing to immune evasion in melanoma and other solid malignancies. Cancer cells and other cells in the tumor microenvironment (TME) can express PD-L1, and PD-L1 levels in the TME strongly predict response to immunotherapy and survival outcomes in patients with cutaneous melanoma (60). Specifically, high PD-L1 expression in the TME was associated with increased objective response rate (ORR) and improved progression-free survival (PFS) and overall survival (OS) rates in patients with melanoma treated with anti-PD-1/PD-L1 monotherapy (61). Notably, PD-L1 expression in the TME is relatively low in acral melanomas. Kaunitz et al. (62) performed immunohistochemical staining for PD-L1 on formalin-fixed paraffin-embedded specimens from patients with melanoma and found that only 31% of acral melanomas expressed PD-L1 in the TME.

Moreover, the poor prognosis of acral melanomas may, to some extent, be attributed to their poor immunogenicity and low mutational burden. Castaneda et al. (63, 64) demonstrated that low numbers of tumor-infiltrating lymphocytes (TILs) were associated with poor prognosis in patients with acral melanoma. CD8+ T cells are particularly important for the elimination of cancer cells, especially in patients undergoing treatment with immune checkpoint inhibitors (ICIs). Edwards et al. (65) showed that CD103+ tumor-resident CD8+ T cells expanded significantly in immunotherapy-naïve patients treated with anti-PD-1 agents (pembrolizumab). They also showed that the levels of tumor-resident CD8+ T cells were more accurate predictors of melanoma-specific survival than the levels of total CD8+ T cells. Interestingly, in a follow-up study, Edwards et al. (66) found that, although the presence of tumor-resident CD8+ T cells was positively associated with OS, TMB and structural variations in acral and mucosal melanomas were not correlated with the densities of tumor-infiltrating CD8+ T cells and other innate and adaptive immune cell types (66).

Comparison of whole-transcriptome and whole-genome sequencing data between Western and Asian melanoma patients indicated that gene signatures associated with antigen presentation and T cell inflammation were expressed in lower levels in Chinese patients than in Western patients (31, 67, 68). Moreover, comprehensive molecular analyses of melanomas from 152 Asian patients (58 with recurrent or metastatic acral melanoma) revealed a significant association between a high neutrophil-lymphocyte ratio (NLR) and the poor survival outcomes of Asian patients undergoing treatment with PD-1 inhibitors (69). The role of neutrophils in the clinical efficacy of ICIs in patients with acral melanoma remains to be determined.

### Current Treatment Options

Evidence on the clinical efficacy of different systemic treatments for acral melanoma from international, large-cohort studies is scarce due to the low incidence rate of acral melanoma. Thus, the establishment of standardized, effective systemic treatments remains an unmet clinical need. In a randomized phase II trial, 158 high-risk Chinese patients with acral melanoma were randomly assigned to receive adjuvant treatment with high-dose IFN-α-2b (HDI) for four weeks or one year. The median relapse-free survival (RFS) for 4-week HDI and 1-year HDI was 17.9 months and 22.5 months, respectively; this difference in RFS was not statistically significant (70). However, the effects of HDI on OS remain controversial (71).

The efficacy of targeted therapies has also been assessed in patients with acral melanoma. Aberrations in MAPK, PI3K/AKT/PTEN, TERT, WNT, and CDK4/CDKN2A signaling pathways are frequent in acral melanoma (43). However, despite the fact that acral melanomas have potentially actionable targets (45), a limited number of targeted therapies are available for the treatment of patients with acral melanoma. The combination of BRAF and MEK inhibitors provides robust clinical responses in patients with *BRAF*-mutated melanoma (72–74). Nonetheless, the frequency of *BRAF* mutations in acral melanoma is relatively low, limiting the clinical benefit of BRAF and MEK inhibitors in this patient population (13, 46). The feasibility of using PI3K/Akt/mTOR inhibitors (75), CDK inhibitors (76), or MDM2/p53 inhibitors (77) to treat acral melanoma is currently being investigated.

Since 2011, various ICIs have been approved by the US FDA and the Chinese National Medical Products Administration (NMPA) for use as monotherapy or in combination with other therapies in melanoma. These agents include ipilimumab, pembrolizumab, and nivolumab (78). The findings of multiple clinical trials indicate that, in Caucasian patients with advanced melanoma, response rates after PD-1 blockade with nivolumab or pembrolizumab range between 26% and 44%. Compared with ipilimumab plus dacarbazine, monotherapy with nivolumab or pembrolizumab provided superior clinical outcomes in patients with advanced melanoma (79–81). However, results from patients with acral melanoma were seldom reported separately in these early clinical studies because of the rarity of this pathological subtype. Only one global, multi-center study (NCT02156804) involved sub-analysis of efficacy in patients with acral melanoma, who represented only 5.5% (n = 55) of the total cohort (**Table 1**). Caucasian patients with acral melanoma had similar survival outcomes to those with non-acral cutaneous melanoma (82). Similarly, in 164 Asian patients with metastatic melanoma, there were no significant differences in clinical responses to ICIs based on melanoma subtypes (59). However, results from clinical trials and retrospective studies are conflicting.

**Table 1:** Summary of immunotherapy clinical trials involving sub-analysis of patients with acral melanoma.

Trial identifier	Phase	Number of patients	Location	Treatment	Line of treatment	Results
NCT02156804	II	n = 1,008 (total) n = 55 (AM)	Europe	Nivolumab	2+	Median OS: 25.8 months (95% CI, 15.1–30.6 months)
NCT02821000	Ib	n = 102 (total) n = 39 (AM)	China	Pembrolizumab	2	ORR, 15.8% (95% CI, 6.0%–31.3%)
NCT03013101	II	n = 128 (total) n = 50 (AM)	China	Toripalimab	2	ORR, 14.3% (95% CI, 5.9%–27.2%)
JapicCTI-142533	II	n = 24 (total) n = 7 (AM)	Japan	Nivolumab	1	ORR, 28.6% (90% CI, 10.0%–59.1%)
JapicCTI-152869	II	n = 30 (total) n = 7 (AM)	Japan	Nivolumab ± ipilimumab	1	ORR, 42.9% (95% CI, 9.9%–81.6%)

AM, acral melanoma; ORR, objective response rate; OS, overall survival.

Shoushtari et al. (83) reported an ORR of 32% [95% confidence interval (CI), 15%–54%] and a median PFS of 4.1 months in 25 patients with acral melanoma after treatment with pembrolizumab or nivolumab. A single-center study of 428 patients with metastatic melanoma (cutaneous in 283, unknown in 55, mucosal in 38, and acral in 22) showed that the median OS of patients with acral melanoma was significantly shorter than that of patients with cutaneous melanoma (17 months *vs*. 45 months; *P* = 0.047) after immunotherapy with CTLA-4, PD-1, or PD-L1 inhibitors (84). Low PD-L1 expression in the TME and low numbers of TILs may contribute to the poor response of acral melanomas to ICIs. The low TMB of acral melanomas may also contribute to the poor outcomes in patients with acral melanomas undergoing treatment with ICIs. Tumors with a high TMB often exhibit superior clinical responses to ICIs *versus* tumors with a low TMB (85, 86). A higher TMB leads to a higher load of neoantigens that may elicit antitumor immune responses during immunotherapy with ICIs (87, 88).

Importantly, increasing evidence suggests that Asian patients with acral melanomas have even lower rates of response to ICI than their Caucasian counterparts. A recent retrospective study of 193 Japanese patients with advanced acral melanoma (palm and sole, n = 123; nail apparatus, n = 70) showed that PD-1 blockade with nivolumab or pembrolizumab provided ORRs of 21.1% in the palm and sole group and 8.6% in the nail apparatus group; median OS was 22.3 months and 12.8 months, respectively (89). The study also showed that the ORR in the palm and sole group was higher in the *BRAF* wild-type group than in the *BRAF*-mutant group (20.7% *vs*. 8.1%; *P* = 0.04) (89). Similar findings were reported in the Chinese cohort of the open-label, non-randomized, multicenter KEYNOTE-151 study evaluating the safety and efficacy of pembrolizumab (90). The KEYNOTE-151 study showed that in 103 Chinese patients with advanced or metastatic melanoma (39 with acral melanoma) previously treated with one line of therapy, pembrolizumab provided an overall ORR of 16.7% (95% CI, 10.0%–25.3%), a median PFS of 2.8 months (95% CI, 2.7–3.5 months), and a median OS of 12.1 months (95% CI, 9.6 months–not reached) (90). In a multicenter phase II study evaluating the safety and efficacy of the humanized anti-PD-1 antibody toripalimab in 128 Chinese patients with advanced melanoma (POLARIS-01), the ORR in 50 toripalimab-treated patients with acral melanoma was 14.0%, the median OS was 16.9 months, and the median PFS was 3.2 months (67). Additionally, *NRAS* mutations and *CCDN1* amplifications, which are particularly common among Chinese patients with melanoma, were associated with poor response to toripalimab treatment (ORRs of 6.3% and 0%, respectively). In a recent study, Byeon et al. (59) reported an overall response rate of 43.1% in 65 Asian patients with acral melanoma after first-line or second-line treatment with ICIs (pembrolizumab, nivolumab, or ipilimumab). These reported clinical outcomes in Asian patients in response to ICIs are considerably worse than those reported in Western populations (83, 91, 92).

Given the role of the TMB and tumor immune microenvironment in clinical responses to immunotherapy and targeted therapies, the molecular differences between melanomas in Asian and Caucasian patients may be critical drivers of any disparities in treatment response between these populations. In addition, genetic alterations in CDK4 pathway components (*CDK4*, *CCND1*, and *CDKN2A*), which are particularly common in Asians, have been associated with innate resistance to PD-1 blockade in patients with acral melanoma (93). Consistently, a recent panel-based next-generation sequencing analysis by Hilke et al. (94) showed that *CDKN2A* deletions or loss of heterozygosity, which are commonly found in non-Caucasian patients with melanoma, were significantly enriched in patients with progressing melanomas after treatment with ICIs (nivolumab, pembrolizumab, or nivolumab plus ipilimumab).

## Mucosal Melanoma

### Clinicopathological Characteristics and Patient Survival

Mucosal melanomas arise from melanocytes in mucosal tissues, such as those in the nasal cavity, the mucous membrane lining the sinuses and mouth, the anus, and the vagina. More rarely, mucosal melanomas may also develop in the gastrointestinal tract, urinary tract, and gall bladder (95). Due to their anatomical location, mucosal melanomas are often diagnosed at an advanced stage, contributing to their aggressive phenotypes and dismal prognosis (95, 96). Compared with cutaneous melanomas, mucosal melanomas present more aggressive characteristics, including advanced TNM stage at diagnosis, high growth rate, and high metastatic potential (97, 98).

In the US, the most common anatomical location of mucosal melanomas is the head and neck (31%–55%), followed by the anorectum (17%–24%) and the vulvovaginal (18%–40%) regions (19). Mucosal melanomas less frequently arise on the mucosal lining of the pharynx, larynx, urinary tract, cervix, and esophagus (16, 19). In a retrospective analysis of 706 Chinese patients with mucosal melanomas, Lian et al. (99) found that the lower gastrointestinal tract was the most common site of primary lesions (26.5%), followed by the nasal cavity and paranasal sinuses (23%), gynecological sites (22.5%), oral cavity (15%), urological sites (5%), and the upper gastrointestinal tract (5%). However, differences in the subclassification system based on the tumors’ anatomical location complicate the comparison of anatomical sites of mucosal melanomas across studies. Lian et al. (99) found that patients with primary mucosal melanomas at the nasal and oral cavities, the gastrointestinal tract, and gynecological and urological sites had similar 1-year (88%, 83%, and 86%), 2-year (66%, 57%, and 61%), and 5-year (27%, 16%, and 20%) OS rates (99). These findings are further supported by a multivariate analysis of the same 706 patients included in the analysis by Lian et al. that found no association between OS and anatomical site, although it did reveal an association between factors including depth of tumor invasion, number of lymph node metastases and sites of distant metastases and OS (100). Interestingly, a population-based study of a racially diverse cohort in California showed that the most common anatomical site of mucosal melanoma was the anorectum in Asian/Pacific Islanders, the genitourinary tract in non-Hispanic Whites, and the head and neck in Hispanics (101). Differences were also observed in the stage of presentation of mucosal melanomas among the different ancestral groups. Notably, 55% of mucosal melanomas in Asians and Pacific Islanders were diagnosed at a metastatic stage; in contrast, the percentage of mucosal melanomas diagnosed at a metastatic stage (locally or distally) was 49% in Hispanics, 47% in non-Hispanic Blacks, and 45% in non-Hispanic Whites (101).

### Mutational Landscape

Like acral melanomas, mucosal melanomas are driven by chromosomal structural aberrations. Their mutational burden is relatively low, perhaps because they are typically not caused by exposure to UV radiation (42, 102–104). A key difference in the melanoma mutational profiles between Asians and Caucasians is that in Caucasians, most melanomas, which are predominantly cutaneous melanomas, are driven by UV-induced point mutations, including driver mutations in *BRAF* and *NRAS* (45). The findings of whole-genome studies suggest that mutations in *SF3B1* are common among mucosal melanomas harboring driver mutations (42, 45). In mucosal melanomas, apart from driver mutations affecting the MAPK pathway (e.g., *NRAS*, *BRAF*, *NF1*, and *KIT*), mutations in *CTNNB1* affecting Wnt/β-catenin pathway activation have also been reported (103, 105). Mutations in Wnt/β-catenin pathway components may contribute to the poor immunotherapy response of patients with advanced-stage mucosal melanoma (106, 107). Notably, Sheng et al. (108) found that nearly 10% of mucosal melanomas in Chinese patients had mutations in either *GNAQ* or *GNA11.* Importantly, these mutations were associated with poor prognosis, likely contributing to the less favorable survival outcomes of Chinese patients with melanoma compared with Caucasian patients (108).

In a recent study of 213 Chinese patients with mucosal melanoma, Xu et al. (109) found that mutations in cell cycle-related genes were particularly common, with 47.0% and 27.7% of samples exhibiting amplifications in *CDK4* and *CCND1*, respectively. Similarly, *P16^INK4^* ^a^ was deleted in 57.7% of mucosal melanomas. Hence, alterations in CDK4 signaling components may predict response to CDK4 inhibitors in patients with mucosal melanoma (109). MicroRNAs may also have a predictive value in mucosal melanoma. Ma et al. (110) identified microRNA-23a-3p as a key tumor-suppressor in mucosal melanoma, inhibiting mucosal melanoma progression by targeting adenylate cyclase 1 (ADCY1) and thereby inhibiting cAMP and MAPK signaling (110).

Similar to acral melanoma, there are limited data describing the association between mutational profiles and response to treatment in mucosal melanoma. However, a study of 178 Asian patients with advanced melanoma (26% with mucosal melanoma) receiving immune checkpoint inhibitors showed that *NRAS* mutations, *TP53* mutations and *NF2* deletions are associated with resistance to checkpoint inhibitors, with *MYC* and *RPS6KB1* amplifications more frequent in patients responding to treatment with immune checkpoint inhibitors (59).

### Tumor Immune Microenvironment

Little is known about the immune microenvironment of mucosal melanoma, although it is believed to be more tolerogenic than that of other melanoma subtypes (18). Similar to acral melanomas, mucosal melanomas express lower levels of PD-L1 in the TME than cutaneous melanomas. Kaunitz et al. (62) demonstrated that, although 62% of chronic sun-damaged melanomas expressed PD-L1 in the TME, only 44% of mucosal melanomas exhibited PD-L1 expression. Analysis of the TCGA dataset revealed that the transcript levels of PD-L1 in melanoma were lower in Asian patients than in Caucasian patients (111); nevertheless, it remains unclear whether mucosal or acral melanomas in Asian patients express lower PD-L1 levels than those in Caucasian patients. Interestingly, compared with Caucasians, Asian patients with melanoma tend to express lower levels of genes associated with antigen presentation and T cell inflammation (31, 67, 68). Impaired antigen presentation and T cell infiltration may significantly contribute to the inferior response rates and survival outcomes of Asian patients with mucosal melanoma undergoing treatment with ICIs. Intriguingly, a retrospective analysis of 152 Asian patients with recurrent or metastatic melanoma (47 with mucosal melanoma) treated with anti-PD-1 agents indicated that a high NLR in the TME was associated with poor response and patient survival (69). Future studies are required to confirm the role of NLR as a biomarker predicting ICI resistance in mucosal melanoma.

### Current Systemic Treatment Options

Mucosal melanomas are typically detected at an advanced stage due to their challenging anatomic location. Owing to the anatomic constraints of mucosal melanomas and the multifocal growth of the lesions, complete resection and wide negative margins are challenging to achieve, leading to a high recurrence rate following surgical management (18, 112). Chemotherapies have similar effects in cutaneous melanoma and mucosal melanoma but failed to significantly improve outcomes. In a multicenter retrospective study, Yi et al. (113) showed that the OS of patients with mucosal melanoma after first-line treatment with dacarbazine-based chemotherapy was significantly shorter than that of patients with cutaneous or acral melanoma (113). As mucosal melanoma is one of the most common melanoma subtypes in China, several Chinese clinical trials have been conducted to evaluate the safety and efficacy of different regimens in patients with mucosal melanoma. In a randomized phase II study, adjuvant therapy with six cycles of cisplatin (75 mg/m^2^) plus temozolomide (200 mg/m^2^) for resected mucosal melanoma with adventitial invasion or nodal metastases provided a significant survival benefit (114). Yan et al. (115) conducted a randomized phase II study in patients with advanced, previously untreated mucosal melanoma. Although first-line chemotherapy (carboplatin plus paclitaxel) combined with antiangiogenic therapy (bevacizumab) did not significantly improve the ORR compared with chemotherapy alone (19.7% *vs*. 13.2%, *P* = 0.384), median PFS (4.8 *vs*. 3.0 months; HR, 0.461; 95% CI, 0.306–0.695; *P* < 0.001) and median OS (13.6 *vs*. 9.0 months; HR, 0.611; 95% CI, 0.407–0.917; *P* = 0.017) were significantly improved in patients treated with combination therapy (115).

The efficacy and safety of targeted therapies, especially of agents targeting c-KIT, have also been investigated in patients with mucosal melanoma. The frequency of *BRAF* mutations in mucosal melanoma is low (105, 116); hence, most patients with mucosal melanoma do not benefit from BRAF inhibitors alone or in combination with MEK inhibitors (117, 118). On the other hand, activating mutations in KIT are relatively common in mucosal melanoma, being found in approximately 40% of all patients (119). However, in patients with *KIT*-mutated metastatic mucosal melanoma, agents targeting c-KIT failed to provide durable responses (120). In a phase II trial evaluating the efficacy of imatinib in patients with metastatic melanoma [17/24 (71%) patients with mucosal melanoma] harboring activating *KIT* mutations or amplifications, imatinib provided an overall response rate of 29% and an overall disease control rate of 50%. However, disease control rates varied depending on the *KIT* status, with patients with *KIT*-mutant melanoma gaining a higher benefit than those with *KIT* amplification (121). Another phase II trial involving patients with melanoma [12/19 (74%) patients with mucosal melanoma] harboring *KIT* mutations or amplifications showed that nilotinib may benefit patients with *KIT* alterations and whose tumors progressed after treatment with imatinib; however, patients with brain metastasis did not benefit from the treatment (122).

Advances in immunotherapies have provided promising therapeutic approaches for patients with advanced mucosal melanoma. Nevertheless, clinical studies suggest that patients with mucosal melanomas tend to be less responsive to ICIs than those with cutaneous melanomas (**Table 2**). This holds true for both Asian and Caucasian patients (123, 124). In a study of 35 patients with mucosal melanoma, monotherapy with pembrolizumab or nivolumab provided an ORR of 23% (95% CI, 10%–40%) and a median PFS of 3.9 months (83). In a single-center study of 428 patients with metastatic melanoma, Klemen et al. (84) demonstrated that immunotherapy with CTLA-4, PD-1, or PD-L1 inhibitors provided a significantly shorter median OS in patients with mucosal melanoma than in those with cutaneous melanoma (18 *vs*. 45 months; *P* = 0.003). However, in a recent study of 164 Asian patients treated with pembrolizumab, nivolumab, or ipilimumab for metastatic melanoma, response rates in patients with mucosal melanoma (41.9%) were not significantly different from those in patients with acral (43.1%) or cutaneous melanoma (54.7%) (59).

**Table 2:** Summary of immunotherapy clinical trials involving sub-analysis of patients with mucosal melanoma.

Trial identifier	Phase	Number of patients	Location	Treatment	Line of treatment	Results
NCT02156804	II	n = 1,008 (total) n = 63 (MM)	Europe	Nivolumab	2+	Median OS, 11.5 months (95% CI, 6.4–15.0 months)
NCT02821000	Ib	n = 102 (total) n = 15 (MM)	China	Pembrolizumab	2	ORR, 13.3% (95% CI, 1.7%–40.5%)
NCT03013101	II	n = 128 (total) n = 22 (MM)	China	Toripalimab	2	ORR, 0% (95% CI, 0.0%–17.6%)
NCT01844505	III	n = 1,295 (total) n = 79 (MM)	International	Nivolumab ± ipilimumab	1	ORR, 43% with NIVO+IPI *vs*. 30% with NIVO and 7% with IPI
JapicCTI-142533	II	n = 24 (total) n = 6 (MM)	Japan	Nivolumab	1	ORR, 33.3% (90% CI, 11.7%–65.3%)
JapicCTI-152869	II	n = 30 (total) n = 12 (MM)	Japan	Nivolumab ± ipilimumab	1	ORR, 33.3% (95% CI, 9.9%–65.1%)
DeCOG-MM-PAL11-Trial	II	n = 103 (total) n = 7 (MM)	Germany	Ipilimumab	2+	Median OS, 9.6 months (95% CI, 1.6–11.1 months)

IPI, ipilimumab; MM, mucosal melanoma; NIVO, nivolumab; ORR, objective response rate; OS, overall survival.

A post-hoc analysis of three trials (KEYNOTE-001, KEYNOTE-002, and KEYNOTE-006) involving 1567 patients with melanoma (84 with advanced mucosal melanoma) revealed that the ORR in patients treated with pembrolizumab was 19% (95% CI, 11%–29%), the median PFS was 2.8 months (95% CI, 2.7–2.8), and the median OS was 11.3 months (95% CI, 7.7–16.6 months) (125). The 5-year follow-up of 79 patients with mucosal melanoma from the CheckMate-067 study revealed that compared with ipilimumab alone, the combination of nivolumab and ipilimumab resulted in a considerably higher ORR (43% *vs*. 7%), complete response rate (14% *vs*. 0%), and OS rate (36% *vs*. 7%). However, clinical outcomes in patients with mucosal melanoma tended to be worse than those in the intent-to-treat population (126). In addition, these different studies included patients with different baseline characteristics and cannot be easily directly compared head-to-head.

Notably, clinical outcomes in response to ICIs tend to be less favorable in Asian patients with mucosal melanomas than their Caucasian counterparts. The KEYNOTE-151 study demonstrated that in 15 Chinese patients with advanced or metastatic mucosal melanoma, pembrolizumab provided an overall ORR of 13.3% (95% CI, 1.7%–40.5%) (90). The POLARIS-01 study showed that clinical outcomes in 22 Chinese patients with mucosal melanoma treated with toripalimab were very poor; the ORR was 0%, the median OS was 10.3 months, and the median PFS was 1.9 months (67). Similarly, a retrospective analysis of 162 Chinese patients with advanced melanoma (121 with cutaneous and 41 mucosal) showed that the overall median PFS of patients with mucosal melanoma was 13 months; the median PFS in the immunotherapy group was 14 months and in the chemotherapy group was six months (127). In a German phase II study, ipilimumab provided an ORR of 17% in seven patients with metastatic mucosal melanoma, and the median OS was 9.6 months (95% CI, 1.6–11.1 months) (128). A retrospective analysis of 75 German patients with mucosal melanoma showed that ipilimumab provided an ORR of 12.5 (median OS and PFS were not reached) (129). In addition, a pooled analysis of patients with mucosal melanoma from the US, Europe, and Australia revealed an ORR of 23.3% (95% CI, 14.8–33.6) and a median PFS of 3.0 months (95% CI, 2.2–not reached) after treatment with nivolumab (124). Similarly, in 84 patients with advanced mucosal melanoma from North America, Europe, and Australia, pembrolizumab provided an ORR of 19% (95% CI, 11–29), a median PFS of 2.8 months (95% CI, 2.7–2.8), and a median OS of 11.3 months (95% CI, 7.7–16.6) (125). A multicenter retrospective study conducted in France revealed that in 151 patients with mucosal melanoma, treatment with anti-CTLA-4 or anti-PD-1 antibodies provided an ORR of 11.9% (95% CI, 7.2–18.2) and a median OS of 15.97 months (interquartile range, 6.89–27.11 months) (130). Shoushtari et al. conducted a retrospective cohort analysis of 35 patients with mucosal melanoma treated with pembrolizumab or nivolumab in the US. They found that PD-1 blockade provided an ORR of 23% (95% CI, 10–40), a median PFS of 3.9 months, and a median OS of 12.4 months (83).

Differences in the prevalence of mutations affecting Wnt/β-catenin pathway activation among different melanoma subtypes (105) may contribute to the unfavorable outcomes of Chinese patients with mucosal melanoma treated with ICIs. Additionally, the high frequency of *KIT* mutations in Asians (131, 132) may contribute to the low response rates of Asians with mucosal melanoma undergoing treatment with targeted therapies.

## Future Directions for the Treatment of Acral and Mucosal Melanomas: Combination Therapies With ICIs

Despite the significant success of ICI monotherapy in the management of advanced cutaneous melanoma, ICI monotherapy is less effective in patients with acral and mucosal melanoma. The difference in response to ICIs may partially be explained by differences in the TMB and immune microenvironment of the tumor. Thus, novel therapies, especially combination therapies, are needed to improve the long-term outcomes of patients with these two melanoma subtypes. Combination immunotherapies that target various phases of the cancer-immunity cycle may represent a promising strategy to overcome immune escape and prevent immunotherapy resistance. By targeting multiple mechanisms by which tumor cells evade immune surveillance, combination therapies may exert synergistic antitumor effects and improve long-term survival outcomes. Numerous ongoing trials are investigating the efficacy and safety of different combination therapies specifically in patients with acral or mucosal melanoma (**Table 3**).

**Table 3:** Summary of ongoing immunotherapy trials for the treatment of acral and mucosal melanoma.

Trial identifier	Phase	Target population	n	Regimens	Setting	Location
NCT03820986 (LEAP-003)	III	Cutaneous, acral, and mucosal melanoma	660	Pembrolizumab + lenvatinib *vs*. pembrolizumab	First-line	International
NCT03955354	II	Acral melanoma	30	SHR‐1210+ apatinib	First-line	China
NCT03991975	I, II	Metastatic and acral melanoma	42	TQB2450 + anlotinib	Second- or later-line	China
NCT04331093	II	Acral melanoma	40	SHR-1210+ apatinib	Neoadjuvant	China
NCT04277663	III	Acral melanoma	300	IBI310+ IBI308 *vs* IBI308 *vs* HDI	Adjuvant	China
NCT04397770	II	Acral melanoma	40	SHR-1210+apatinib+ temozolomide	First-line	China
NCT03602547	II	Mucosal melanoma	40	Toripalimab+CM082	First-line	China
NCT03241186	II	Mucosal melanoma	36	Ipilimumab+nivolumab	Adjuvant	US
NCT03986515	II	Mucosal melanoma	40	Apatinib+SHR-1210	Second- or later-line	China
NCT04622566	II	Mucosal melanoma	26	Pembrolizumab+lenvatinib	Adjuvant	China
NCT04462965	II	Mucosal melanoma	294	Toripalimab+temozolomide	Adjuvant	China
NCT04318717	I, II	Mucosal melanoma of head and neck	16	Pembrolizumab+ hypofractionated radiotherapy	Adjuvant	US
NCT04180995	II	Mucosal melanoma	30	Toripalimab, axitinib	Neoadjuvant	China
NCT04091217	II	Locally advanced or metastatic mucosal melanoma	43	Atezolizumab+bevacizumab	First- or later line	China
NCT03941795	II	Metastatic mucosal melanoma	99	Toripalimab+axitinib	First-line	China
NCT02978443	II	Metastatic acral and mucosal melanoma	14	Nivolumab+ipilimumab	First- or later line	US
NCT04653038	I	Unresectable, recurrent, or metastatic acral and mucosal melanoma	160	MGD013 (tebotelimab)	Second- or later-line	China

A study of 20 patients with primary mucosal oral melanoma demonstrated that the expression level of vascular endothelial growth factor (VEGF) was associated with poor survival outcomes (133), suggesting VEGF to be a promising therapeutic target for the treatment of oral mucosal melanoma. Nevertheless, no significant improvement in survival outcomes was observed in patients with advanced melanoma treated with anti-angiogenic therapy alone compared with those treated with chemotherapy (134, 135). In addition to its role in neovascularization, VEGF signaling has also emerged as a critical immunosuppressive mechanism in the tumor microenvironment. *In vivo* findings from murine cancer models indicated that the combined inhibition of the VEGF receptor (VEGFR) and PD-1 synergistically increased the number of TILs and inhibits tumor growth (136). Preliminary findings of an ongoing clinical study evaluating the safety and efficacy of axitinib combined with toripalimab, a humanized antibody targeting PD-1, revealed that the combination therapy was tolerable and exhibited encouraging clinical efficacy in 29 treatment-naïve Asian patients with metastatic mucosal melanoma. The ORR was 48.3% (95% CI, 29.4%–67.5%) (137, 138). The authors of this study (138) found no correlation between common driver mutations and clinical response to the combination therapy. However, PD-L1 expression and high TMB were significantly associated with high ORR and PFS, especially in PD-L1 positive patients (138).

Similarly, the phase Ib/II KEYNOTE-146 trial showed that pembrolizumab combined with lenvatinib exerted promising antitumor activity (ORR, 47.6%; 95% CI, 25.7–70.2) in patients with advanced melanoma (139). An international randomized phase III trial (LEAP-003, NCT03820986) of pembrolizumab combined with lenvatinib for the treatment of metastatic melanoma is currently ongoing and includes patients with acral and mucosal melanomas. Furthermore, in a phase II study of patients with advanced mucosal melanoma (NCT03602547) in our center, we found that the combination of toripalimab with the multi-target tyrosine kinase inhibitor vorolanib (CM082; 150 mg) provided an ORR of 22.2%, a disease control rate of 55.5%, and a median PFS of 5.7 months (140). Similar small clinical trials of combination regimens (e.g., ICI combined with anlotinib, apatinib, or bevacizumab) for the treatment of acral and mucosal melanoma are also ongoing.

The efficacy of ipilimumab plus nivolumab in patients with mucosal melanoma is also under investigation. A recent pooled analysis of CheckMate-067 and CheckMate-069 trials showed that, compared with patients treated with nivolumab monotherapy, 35 mucosal melanoma patients treated with the combination of nivolumab plus ipilimumab exhibited higher ORR (37.1% *vs*. 23.3%) and median PFS (5.9 *vs*. 3 months). However, severe toxicities were common among patients receiving the combination therapy (124). Response to ipilimumab plus nivolumab in patients with mucosal melanoma was associated with PD-L1 expression in tumor cells (141). In an open-label, single-arm, multicenter phase II study, first-line nivolumab combined with ipilimumab in Japanese patients with different types of unresectable or recurrent melanoma provided an ORR of 33.3% and a 1-year OS rate of 83.3% (142). Other combination regimens, including ICI combined with temozolomide (an orally active congener of dacarbazine) and radiotherapy (mucosal melanoma of head and neck), have also been initiated or planned.

## Conclusions

Acral and mucosal melanomas share numerous clinicopathological features, including a late onset, aggressive phenotypes, a broad radial growth phase with prominent lentiginous growth, the lack of driver mutations that are common in other melanoma subtypes, and poor prognosis. Despite recent advances in the treatment of melanoma, patients with these rare and aggressive melanoma subtypes show limited benefit from current therapies. Standardized and effective interventions for the treatment of patients with acral and mucosal melanomas are currently lacking. As acral and mucosal melanomas are highly aggressive, combination therapies are more likely to provide long-term clinical benefit. The combination of anti-angiogenic therapies with ICIs is among the most promising strategies to suppress the progression of acral and mucosal melanomas and improve long-term survival outcomes. The clinical efficacy and safety of such combination approaches are currently under extensive clinical investigation. Given the rarity of these melanoma subtypes, large multicentric prospective clinical trials are warranted to improve long-term outcomes in patients with acral and mucosal melanomas, the predominant pathological subtypes of melanoma in Asians and people of African ancestry.

Mounting evidence shows that, compared to patients with cutaneous melanomas, those with acral and mucosal melanomas respond less well to current and emerging immunotherapies and other systemic treatments. Furthermore, ancestral disparities in melanoma treatment outcomes and patient survival also seem to exist. However, evidence for these ancestry-related differences in melanoma treatment outcomes is mostly derived from retrospective population-based studies or comparisons of results from various small-cohort clinical trials involving patients that did not necessarily have the same baseline characteristics. Hence, well-designed clinical trials involving baseline characteristic-matched patients with melanoma are required to confirm the ancestral disparities in acral and mucosal melanoma treatment outcomes. Enhancing the enrollment of racial minorities in clinical trials may also help develop effective therapies to treat aggressive melanoma subtypes that are rare among Caucasian patients.

Additionally, differences in the clinicopathological features, mutational profiles, and tumor immune microenvironments may contribute to disparities in melanoma treatment outcomes; hence, these factors should be taken into account in clinical decision making. Understanding the genetic and environmental determinants of melanoma disparities is paramount to facilitating early diagnosis, developing effective treatments, and improving survival outcomes in patients with acral and mucosal melanoma.

## Author Contributions

LM, ZQ, LZ, JG and LS contributed to the conception of the review, conduct of the literature review, manuscript drafting and approval of the final draft. LZ collected or assembled the data and provided substantive suggestions for revision or critically reviewed subsequent iterations of the manuscript. All authors contributed to the article and approved the submitted version.

## Funding

This review was funded by MSD China.

## Conflict of Interest

JG is a member of the advisory board/consultant of MSD, Roche, Pfizer, Bayer, Novartis, Simcere, Shanghai Junshi Bioscience, Oriengene. LZ is an employee of MSD China.

The authors declare that this review received funding from MSD China. The funder had the following involvement in the review: formally reviewing a penultimate draft.

The remaining authors declare that the research was conducted in the absence of any commercial or financial relationships that could be construed as a potential conflict of interest.
